# Supplementary material for: Laser Cutting Coupled with Electro-Exfoliation to Prepare Versatile Planar Graphene Electrodes for Energy Storage
Source: Int J Mol Sci. 2023 Mar 15;24(6):5599. doi: 10.3390/ijms24065599 (PMC10059886; doi:10.3390/ijms24065599)
Supplement: Supplementary file 1 [file ijms-24-05599-s001.zip › ijms-2264375-supplementary.pdf]

Supplementary data

Laser Cutting Coupled with Electro-exfoliation to Prepare Versatile Planar Graphene Electrodes for Energy Storage

Jianren Wang [1,2], Tianshuo Yang [2], Neus Vila [1] and Alain Walcarius [1,*]

[1] Université de Lorraine, CNRS, Laboratoire de Chimie Physique et Microbiologie pour les Matériaux et l'Environnement (LCPME), F-54000 Nancy, France; wangjr@ysu.edu.cn (J.W.); neus.vila@univ-lorraine.fr (N.V.)

[2] Key Laboratory of Applied Chemistry, School of Environmental and Chemical Engineering, Yanshan University, Qinhuangdao 066004, China

** Correspondence: alain.walcarius@univ-lorraine.fr; Tel.: +33-3-72-74-73-75*

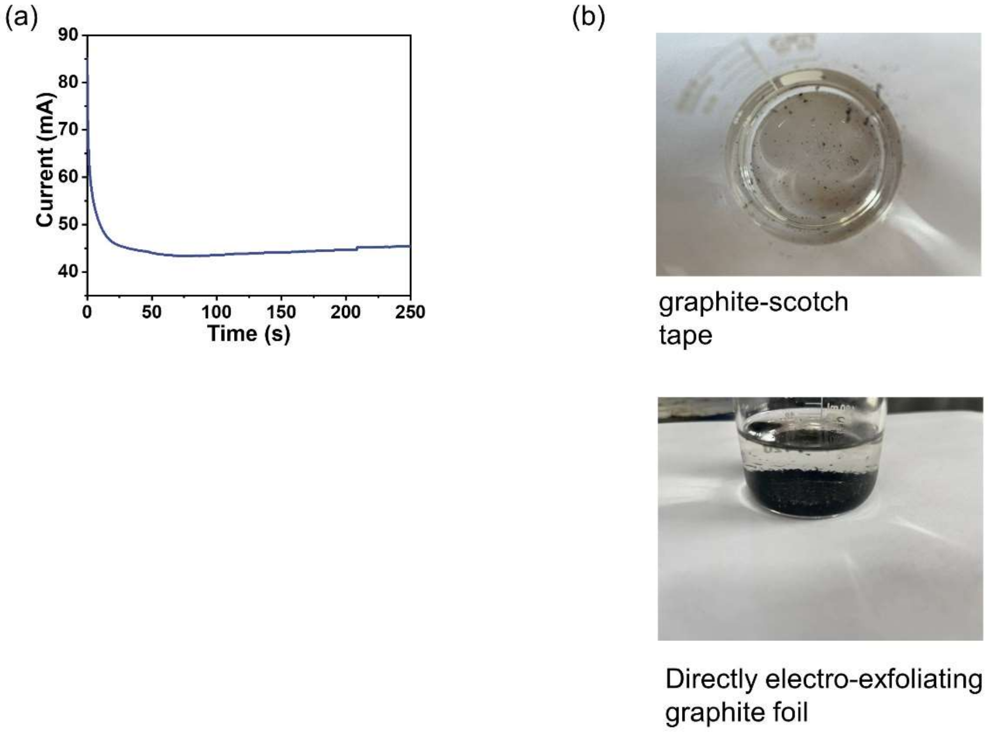

Figure S1 (a) a typical exfoliation current for a piece of graphite foil; (b) Respective photographs of the final solution that exfoliates graphite-scotch tape and a piece of graphite under a same condition.

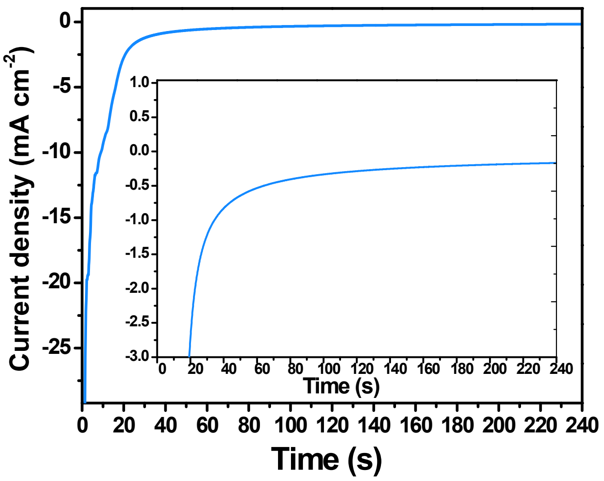

Figure S2 the activation current profile of the G-240 at the potential of 0V for 240s.

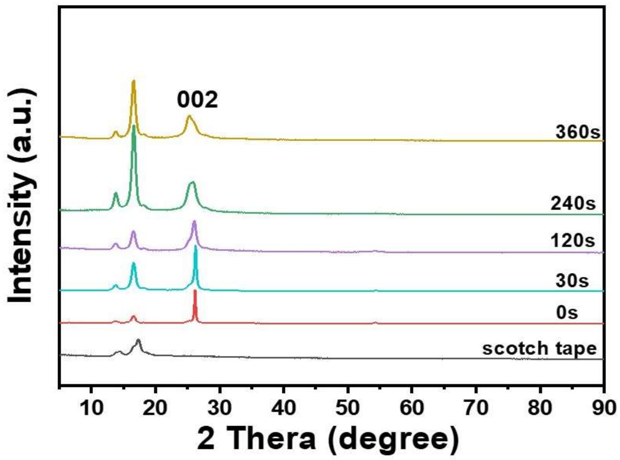

Figure S3 the XRD pattern of the set of graphite samples exfoliated at various times.

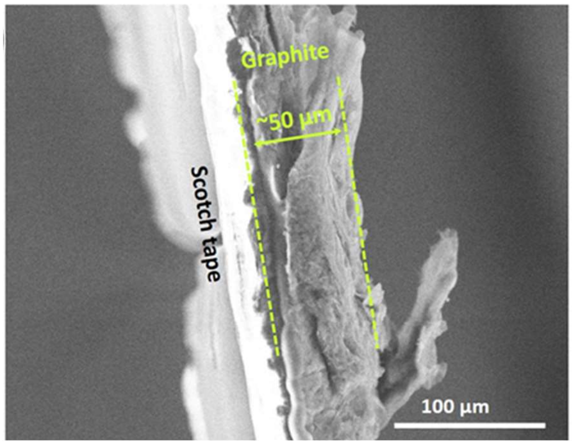

Figure S4 the cross-section SEM view of the original graphite/scotch tape electrode.

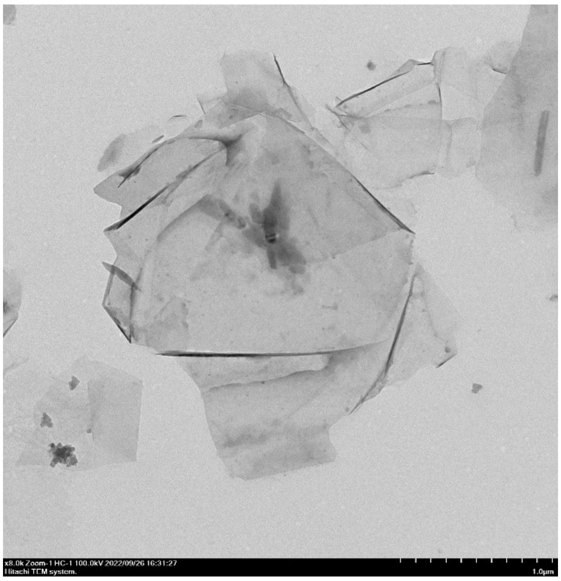

Figure S5 the TEM image of the G-240.

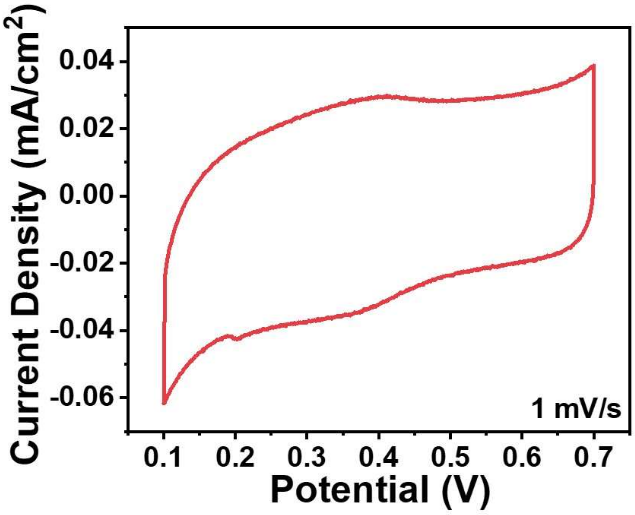

Figure S6 the CV curve of the G-240 at a scan rate of 1 mV s^{-1} in 1M H_2SO_4 with a reference of Ag/AgCl (3 M).

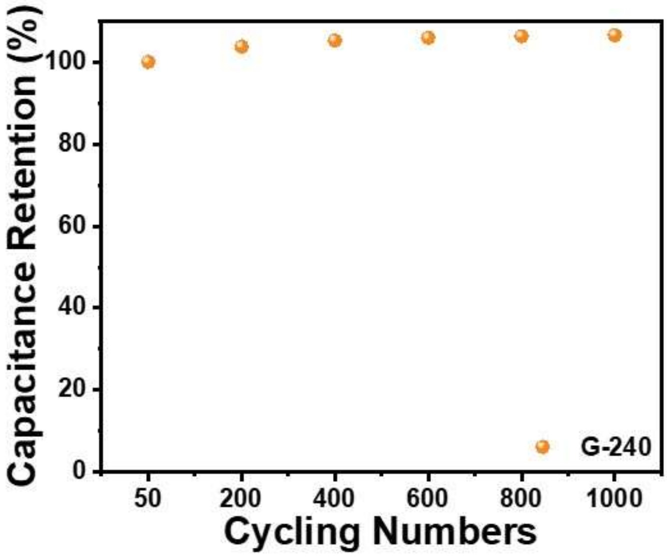

Figure S7 the cycling performance of G-240 at a scan rate of 100 mV s^{-1}.

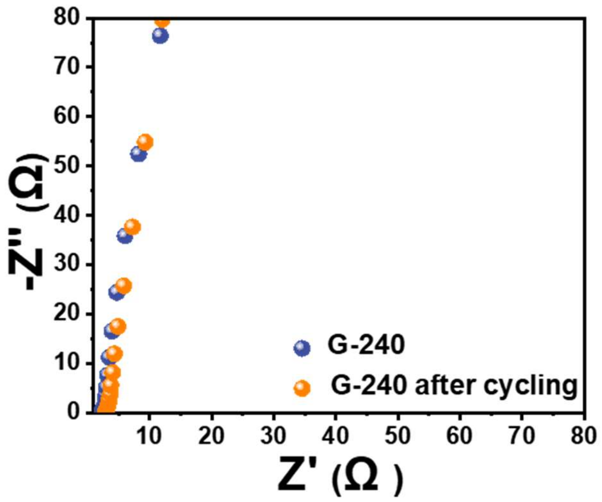

Figure S8 the impedance spectra of G-240 and G-240 after long-term cycling at OCP in the frequency range from 100 kHz to 0.1 Hz.

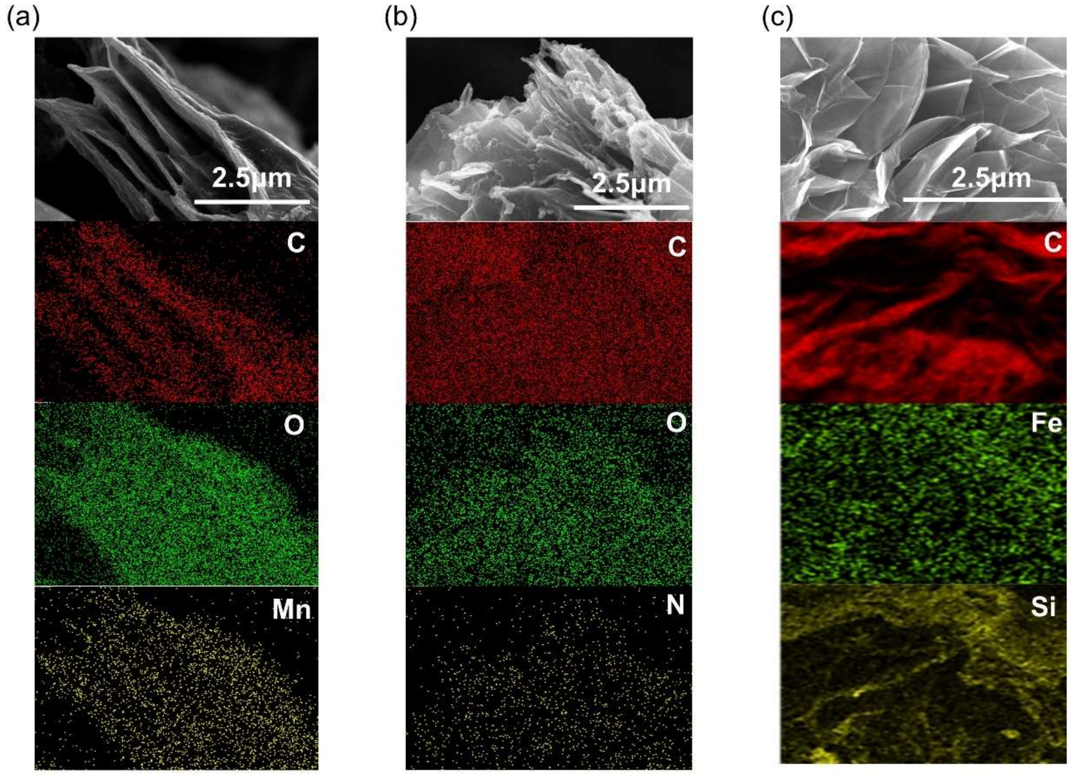

Figure S9 the corresponding elements mapping results of (a) MnO_2, (b) PANI, and (c) Fc-MS functionalized samples to show their homogeneous distribution.

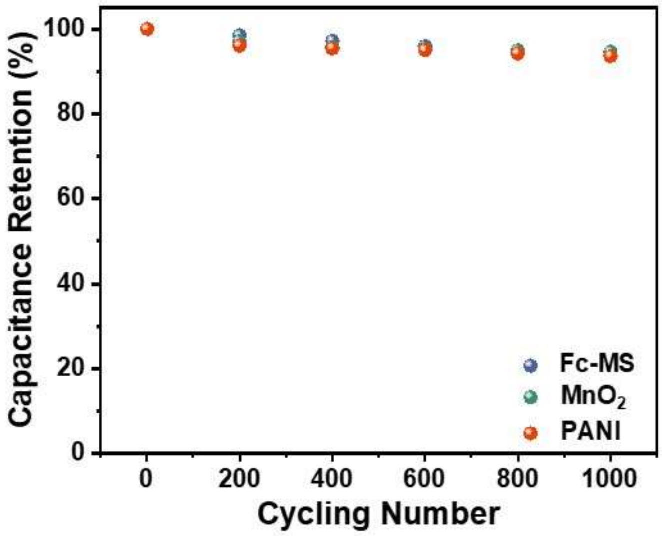

Figure S10 the cycling performance of Fc-MS, MnO_2, and PANI at a scan rate of 100 mV s^{-1}.

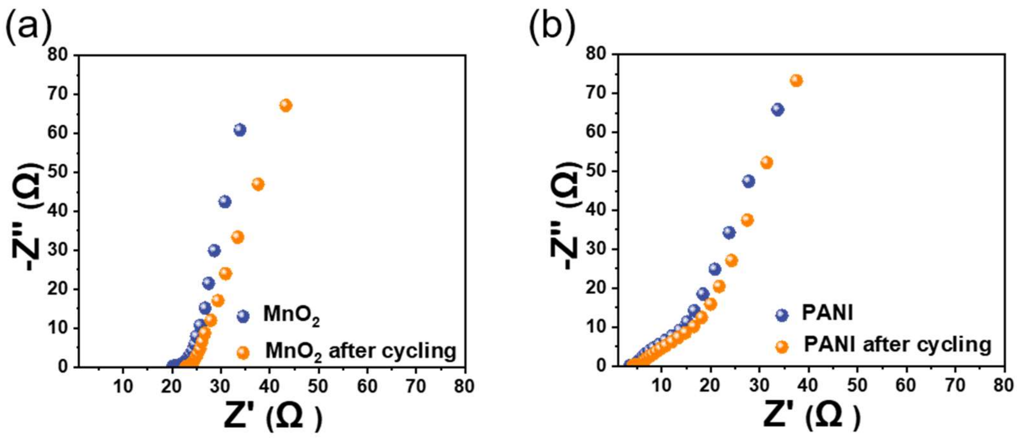

Figure S11 the impedance spectra of MnO_2 (a) and PANI (b) before and after long-term cycling at OCP in the frequency range from 100 kHz to 0.1 Hz.

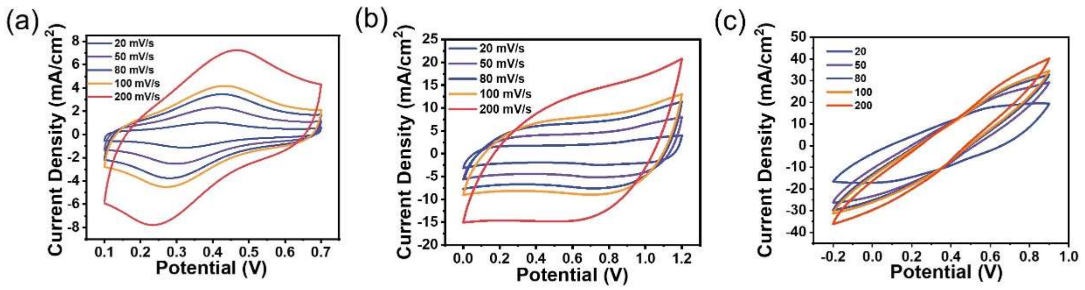

Figure S12 the CV curves of Fc-MS (a), MnO_2 (b), and PANI (c) at scan rates ranging from 20 mV s^{-1} to 200 mV s^{-1} (*vs.* Ag/AgCl 3 M).

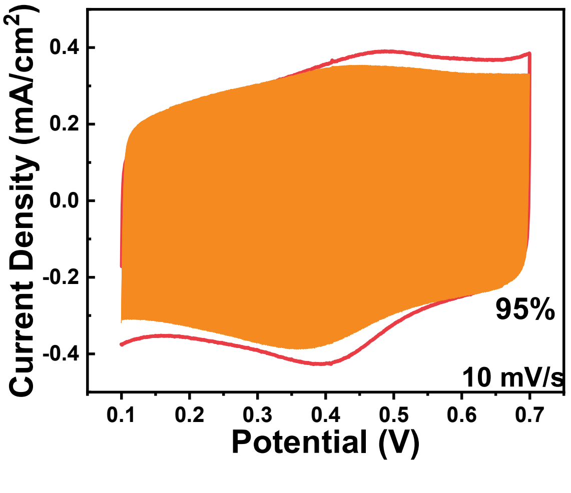

Figure S13 the surface-controlled current ratio of G-240 at a scan rate of 10 mV s^{-1}.

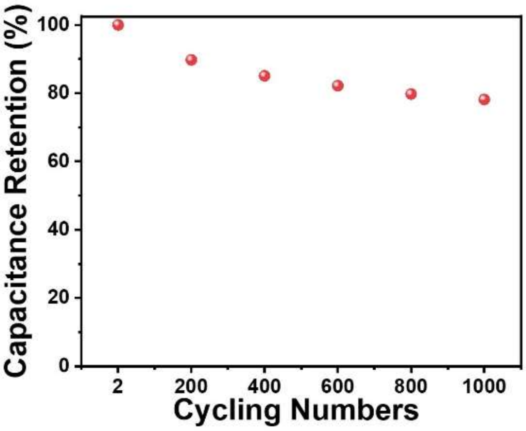

Figure S14 the cycling performance of the PANI symmetrical device.

Table S1 the impedance fitting parameters of the graphite, G-240, and G-240 before activation.

Samples	R_s/Ω	$Q_1/F{*}s^{(a1)}$	a_1	R_{ct}/Ω	$s_2/\Omega{*}s^{(-0.5)}$	Equivalent circuits
graphite	0.7048	0.06246	1		16.18	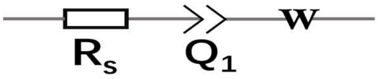
G-240	2.216	0.02161	0.9831		2.679	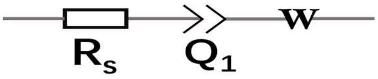
G-240 before activation	1145	$7.654*10^{-5}$	0.3253	7703	$6.547*10^{-12}$	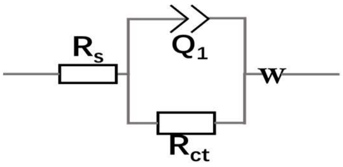
